# The Role of Comparative Psychology in the Training of Veterinarians

**DOI:** 10.3390/ani13142315

**Published:** 2023-07-14

**Authors:** Brooke A. Boughton, Charles I. Abramson

**Affiliations:** Laboratory of Comparative Psychology and Behavioral Biology, Oklahoma State University, Stillwater, OK 74078, USA; bbought@okstate.edu

**Keywords:** comparative psychology, veterinary education, teaching

## Abstract

**Simple Summary:**

Comparative psychology is the oldest of the social sciences, and is one of the natural science branches of psychology. This article is the first to suggest that comparative psychology be incorporated into the vet school curriculum. Many aspects of comparative psychology form natural links with the training of veterinary students. These links include the study of ethics, animal behavior, research methodology, and animal welfare.

**Abstract:**

This article highlights some of the advantages that comparative psychology offers the veterinary student and veterinary education generally. Comparative psychology is the oldest of the social sciences and, as such, has accumulated over three centuries of experience in such areas as research design, animal–human interactions, and animal behavior. To establish whether comparative psychology is taught in veterinary schools, we survey all course catalogs of U.S. veterinary schools. None of the schools surveyed offered a course in comparative psychology, and inconsistencies were noted among the schools in regard to courses in animal–human interaction, animal behavior, and ethics. Suggestions are provided on how to incorporate principles of comparative psychology in veterinary education at both the undergraduate and graduate levels.

## 1. Introduction

One of the most important challenges facing veterinary science is the training of veterinary students. In the United States (U.S.), the veterinary curriculum is usually four years, at the end of which the student receive the degree of Doctor of Veterinary Medicine. Throughout the four years, students receive training in a variety of areas, including anatomy, animal–human interaction, biochemistry, comparative anatomy and physiology, endocrinology, ethics, neurobiology, nutrition, parasitology, pharmacology, and a host of other specialized courses.

The purpose of this article is to recommend that veterinary students take a course at either the undergraduate or graduate level in comparative psychology. Comparative psychology (CP) is the oldest of the organized social sciences, with the term first used in 1778 [1]. As the oldest of the social sciences, CP has the most experience of any behavioral science working on the fundamental issue of animal behavior including ethics, animal–human interaction, and experimental design [2,3].

CP has been defined as the “application of the comparative method to problems in psychology” [4,5]. As such, it is a general psychology with methods and results applicable to both human and non-human animals. Indeed, one of the goals of CP is to conduct a variety of comparisons of humans with non-human animals. CP is one of the few natural science branches of psychology and is arguably the only behavioral science that specifically compares human and non-human animals. As such, we believe that many of the principles, methods, and data of CP are a natural fit with veterinary education. As far as we can determine, the present article is the first to suggest that a link between veterinary education and a course in comparative psychology would be beneficial to veterinary students.

CP differs from fields such as animal behavior and comparative cognition. While some aspects of CP may overlap with other animal behavior courses/disciplines, they are not identical [5]. Students of CP receive combined training in the principles of behavior and methods of comparative analysis. Of relevance to veterinary education is that CP courses contain components related to animal–human interactions, the use of conditioning methods, and the role of animals in the therapeutic process. Seldom, if at all, are these topics covered in any other course that focuses on animal behavior. Moreover, in the best CP courses, students receive laboratory experiences where they directly compare the behavior of various animals, including humans.

We believe that CP has much to recommend for veterinary training. First, as a general psychology, CP offers students a variety of perspectives, many of which are directly related to veterinary training. These include a focus on ethics, animal–human interaction, the use of animals as a therapeutic adjunct, and the use of clinical psychological methods to treat animals for depression and other behavioral ailments. Moreover, as CP is psychology-based, students also receive instruction in the more social aspects of dealing with animals and interacting with fellow humans. Recently, it has been suggested that an interaction between CP and clinical psychology would be beneficial to both fields [6]. This emphasis on the social interactions of human animals is perhaps unique to psychology-based courses.

Second, a course on CP contains information on how to use classical and operant conditioning methods to train a variety of animals in a variety of situations. For instance, conditioning methods are routinely used not only to increase the production of farm animals, but also to improve their welfare [7,8]. Moreover, the training of companion animals is also based upon some form of conditioning. The fact that veterinary students have little to no theoretical and practical experience in classical and operant conditioning puts them at a distinct disadvantage when confronted with the behavioral problems of their clients.

Third, the research designs used in CP will help the veterinary student better understand good research, and recognize poor and misleading veterinary research. For those veterinary students interested in conducting research, a thorough understanding of comparative research methods will dramatically increase the reliability of their findings [2]. Even a cursory understanding of the research methods used in CP will help the student better understand research, and make them better researchers and consumers of veterinary science.

Consider, for example, that one of the most fundamental research methods in CP is the ethogram (also known as a behavioral profile). A veterinarian may be confronted with a situation where the human caregiver may state that their pet “always engages in the problem behavior”. A veterinarian with a background in CP will immediately know that this is not true, as there are “behavioral patterns”. The veterinarian will then request that the human caregiver keep a detailed record of, for example, the time of occurrence, the intensity of occurrence, and what was happening the moment the problem behavior was expressed. In effect, the human client becomes a citizen scientist [9]. With such a detailed record, both the veterinarian and the human caregiver are in a better position to treat the behavioral problem. A Google search will reveal that there are literally hundreds of ethograms on all aspects of animal behavior that the veterinarian can refer to. Moreover, there are videos on how to prepare an ethogram. The senior author (CIA), for example, co-developed an ethogram to evaluate enrichment devices for elephants [10].

Fourth, because CP contains a significant social science aspect, the veterinary student will be in a better position to understand the social bond between the animal and the human caregiver. As mentioned earlier, animal–human interaction is a significant research area in CP. No other psychology or animal behavior course maintains such a focus.

## 2. Search Methodology

To determine if CP is currently covered in veterinary schools, we looked at the course catalogs of all 32 veterinary schools in the United States (U.S.). We restricted our search to U.S. veterinary schools, as we could not access data from non-U.S. schools. We looked at three areas covered in a typical CP course to determine whether some aspects of CP are covered. These areas are animal welfare, animal behavior, and ethics. The results are below, and clearly show that these fundamental aspects of CP are seldom, if at all, included in the veterinary school curriculum.

All university course catalogs were accessed and evaluated (omitting the University of Florida, because student login credentials were required). Courses in welfare, ethics, and behavior were all recorded on an Excel spreadsheet. Course numbers and course descriptions, as well as whether the course was offered as an elective or core class, were also recorded in the spreadsheet.

## 3. Results

The data we obtained from our search of veterinary school catalogs is presented below. The catalogs were examined from November 2022 to January 2023. We believe they are the most current catalogs at the time this article was submitted. The first graph represents the number of schools offering courses related to animal welfare. The second graph represents the number of schools offering courses in animal behavior. The last graph represents the number of ethics-related courses offered by veterinary schools.

**Figure 1:**
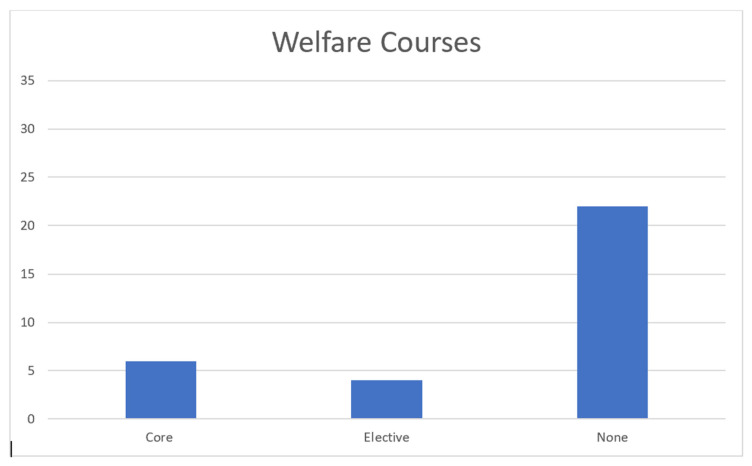
Distribution of animal welfare courses for schools of veterinary medicine.

Animal Welfare Course Data: Figure 1 shows that, of the 32 AVMA-accredited veterinary colleges that allowed access to course catalogs, six (19%) required an animal welfare course to be taken by the veterinary students. Only four (12%) colleges offered a welfare class as an optional elective course, and the remaining 22 (69%) colleges do not offer a welfare course.

**Figure 2:**
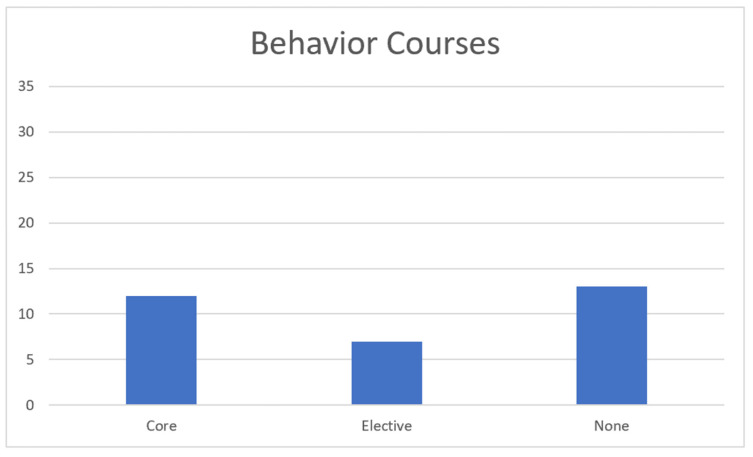
Distribution of animal behavior courses for schools of veterinary medicine.

Behavior Course Data: Figure 2 shows that, of the 32 AVMA-accredited veterinary colleges that allowed access to course catalogs, 12 (37%) required a behavior course to be taken by the veterinary students. Seven (22%) colleges offered a behavior course as an optional elective, and the remaining 13 (41%) universities do not offer a behavior course.

**Figure 3:**
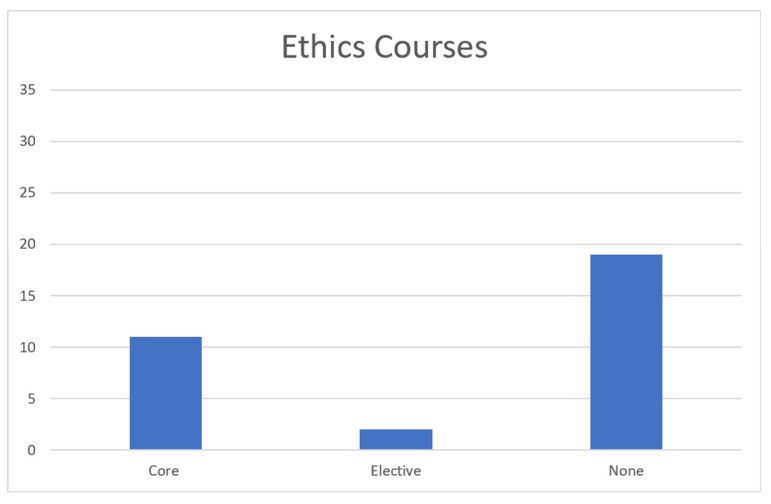
Distribution of animal ethics courses for schools of veterinary medicine.

Ethics Course Data: Figure 3 shows that, of the 32 AVMA-accredited veterinary colleges that allowed access to course catalogs, only 11 (35%) require a core class in ethics to be taken. Two (6%) of the colleges offer ethics as an optional elective course, and the remaining 19 (59%) do not offer any sort of ethics course.

## 4. Resources

It is beyond the scope of this paper to provide specific exercises that can be incorporated into a veterinary school curriculum. Many resources can be found in the open-access journal *The International Journal of Comparative Psychology* (*IJCP*) (https://escholarship.org/uc/uclapsych_ijcp accessed on 3 July 2023). In addition to free access to articles related to CP, *IJCP* recently published two special issues related to teaching (Volume 33) and the state of CP (Volume 31), respectively. These two special issues will be particularly important for those educators wanting to either develop a CP course specifically designed for veterinary students and/or incorporate elements of CP into an existing course. These resources can also serve as independent readings, and as a base for discussion at the administrative level for those programs considering integrating CP into their curriculum on a formal basis.

The special issue related to teaching (https://escholarship.org/uc/item/78468853 accessed on 3 July 2023) includes 12 papers summarizing over 50 classroom-tested inquiry-based exercises. A wide variety of invertebrates and vertebrates are used, ranging from tardigrades to humans. The activities range from experiments on basic conditioning to the construction of ethograms. Of particular interest are papers describing how to construct apparatus using 3D printers, and how to construct low-cost robotic animals [11].

The current state of comparative psychology is the focus of the second special issue [12]. This issue contains 13 articles that cover a wide range of topics. These topics include comparative methodology, data analysis, equine therapy, and the philosophy behind CP (https://escholarship.org/uc/item/0mn8n8bc accessed on 3 July 2023). One paper provides general resources including books, review articles, history, websites, and teaching exercises [5].

## 5. Benefits of CP to Both Undergraduate and Graduate Veterinary Students

Incorporating CP at the graduate level offers veterinary students several advantages. Many of these we have already mentioned, including the study of ethics, the implementation of research design, and the use of conditioning methods. Moreover, the “social” nature associated with a psychology course will better prepare students for interacting with the public and, perhaps, better understanding themselves.

As just one example, consider how conditioning methods are used to improve the lives of farm animals and to increase their production. Abramson [13] shows how classical and operant conditioning is used to modify the behavior of farm animals, and Baldwin [7] reports on how operant techniques are used by animals to regulate their environment. Functional knowledge of conditioning methods is not only useful for veterinarians working with hoofed animals, but also for those working with companion animals exhibiting behavior problems.

Moreover, conditioning methods can be used to improve the lives of animals by reducing environmental stress. In an unpublished experiment, the senior author (CIA) trained a llama to poke its head through a “hula hoop” for a food reward. Head poking was automatically recorded, and a food bin was programmed to operate when the llama met specific contingencies. This method, which was later adapted for horses [14], was subsequently used by the llama to tell the researcher which food it preferred, to turn on and off a water, spray, and to turn on and off a fan. The latter two rewards were particularly useful in the hot Oklahoma summers, where temperatures can reach in excess of 100° Fahrenheit (37.8° Celsius).

For veterinarian students focusing on research, there is no behavioral science that has more experience designing and interpreting animal behavior experiments than CP [2]. Consider the following example. The senior author, who is a comparative psychologist (CIA), was asked to join a team to evaluate the digestive properties of dog chews such as Greenies^TM^. Rather than test the product on a dog, he modified the original method of Pavlov [15] by placing the digestive juices in a test tube along with the product. The degradation of the product could now easily be determined by weighing the remaining sample as a function of time. This procedure has the added advantage that it permits the parametric manipulation of several independent variables, such as the amount of gastric juices. This procedure eliminated any possible stress in test animals, as none were used [16].

Other examples where the methods of CP were used to gather data of interest to veterinarians include a study on the use of silver vine (*Actinidia polygama* Maxim) as an enrichment aid for cats [17], the development of a survey instrument to match a human’s personality characteristics to those of their pets [18], issues related to equine therapy [19], an analysis of reproductive endocrinology and musth in a captive Asian elephant [20], a comparison of food vs. human contact as a reward for horses [21], and a study investigating the ability of horses to measure time [14].

The above examples focus on traditional research areas familiar to veterinarians. However, an understanding of CP can be used in areas not normally considered by veterinarians. These include the relationship of CP to pet industry litigation [22], and how consumer products affect animal behavior [23].

At the undergraduate level, we would encourage pre-vet majors to take a course in comparative psychology. Such a course, in combination with those typically required for a pre-vet major, will significantly enhance their preparation for graduate training. The results of our survey of veterinary schools show the graduate training of veterinary students in the area of ethics, behavior, and animal welfare is not consistent. These topics are part of any competent undergraduate course in CP.

## 6. Limitations and Challenges

This article advocates incorporating CP in the training of veterinarians at the graduate and at undergraduate levels. We also suggest that CP be incorporated into the training of veterinary professional associates (VPA). The development of VPA fills a much-needed gap in veterinary practice [24]. We would advocate that those veterinarian policymakers charged with developing VPA programs consider adding a course in comparative psychology.

Despite the many advantages of CP [3,4,5], there are challenges associated with finding CP programs. A major challenge is that few psychology departments associated with universities that maintain veterinary schools offer CP courses to their undergraduates. In our previous data set, we omitted the University of Florida because a login was required. No such restriction was present to access the course listing in the psychology department. Of the 33 total AVMA-accredited veterinary colleges in the United States, seven (21%) offered comparative psychology courses to their undergraduate students, and seven (21%) additional universities offered an animal behavior course, but did not include comparative psychology in the course title. The remaining 19 (58%) universities did not offer any comparative psychology or animal behavior courses to their undergraduate students.

The situation is perhaps more dire when graduate programs in CP are considered. Of the 33 total AVMA-accredited veterinary colleges in the United States, not a single university offers a strictly CP program. Eight (24%) universities offer an animal-behavior-focused graduate program, and the remaining 25 (76%) universities do not offer any CP or animal-behavior-focused graduate programs.

The lack of CP programs is not restricted to universities that maintain veterinary schools. The problem is nationwide. Of the 650 universities and colleges surveyed, including those that maintain veterinary schools, only 82 (12%) offered an undergraduate course in CP [5]. This absence of CP programs is reflected by the fact that there is only one currently available textbook [25].

## 7. Recommendations and Discussion

As mentioned in the Introduction, we believe this is the first article to suggest that comparative psychology be incorporated into the veterinary school curriculum. To provide some supporting evidence for our assertation, we looked at volumes 25 (2001) through 50 (2023, numbers 1 and 2) of the *Journal of Veterinary Medical Education*. Discounting, letters to the editor, editorials, and book reviews, of 1,360 articles, only three fall into the general area of CP [26,27,28].

Despite the limitations and challenges, we believe that incorporating CP into the veterinary school curriculum will benefit both CP and the teaching of veterinary students. Given the lack of comparative psychology courses and graduate programs, we suggest that those interested in incorporating CP:

1. Work closely with a comparative psychologist to develop curricula. The International Society for Comparative Psychology operates a Listserv (http://www.comparativepsychology.org/accessed on 3 July 2023) and Division 6 (Society for Behavioral Neuroscience and Comparative Psychology) of the American Psychological Association (APA) maintains a list of comparative psychologists. Moreover, the APA has an educational consulting service consisting of individuals specializing in evaluating and developing educational resources;
2. Commission a CP textbook specifically designed for veterinary students;
3. Develop an online CP course for veterinary students;
4. Integrate CP courses into the undergraduate pre-vet curriculum;
5. Assign CP readings for veterinary students without access to CP programs;
6. Hire comparative psychologists as faculty members/consultants;
7. Design an intensive one-week “mini-course” in comparative psychology.

Although the data and ideas discussed in this paper are based on U.S. institutions, we believe that the situation is similar in other parts of the world. Several papers have been written advocating for CP in Africa [29,30] and Asia [31]. These papers, and the present contribution, can help colleagues in these parts of the world better understand CP, and stimulate them to incorporate CP into their countries’ veterinary training.

In conclusion, we believe that, with some effort, CP can significantly add to the training of veterinarian students.

## 8. Conclusions

The partnership between comparative psychology and aspects of veterinary training seems a natural one. Both have a stated interest in the ethical treatment of animals, a focus on behavior, and a concern for animal welfare. Moreover, both share an interest in animal–human interactions and the use of behavioral techniques. Behavioral techniques are used to treat problem behaviors and to increase the output of farm animals, by establishing appropriate conditions of reward and designing suitable environments conducive to production. Moreover, the research designs associated with CP are some of the oldest available, and help the veterinarian student plan better experiments and recognize good behavioral science.

Integrating CP into veterinary school curricula will not be easy. Few veterinary schools have associated psychology departments offering CP courses. There are also few graduate programs. We have provided several suggestions, such as independent readings, developing a CP textbook designed for veterinarian students, and creating an online course.

## Data Availability

The data presented in this study are available on request from the corresponding author.
